# Multi-centric study for development and validation of a CT head rule for mild traumatic brain injury in direct oral anticoagulants: the HERO-M nomogram

**DOI:** 10.1186/s12873-023-00884-w

**Published:** 2023-10-15

**Authors:** Naria Park, Greta Barbieri, Gianni Turcato, Alessandro Cipriano, Arian Zaboli, Sara Giampaoli, Antonio Bonora, Giorgio Ricci, Massimo Santini, Lorenzo Ghiadoni

**Affiliations:** 1 Emergency Medicine Department, Pisa University Hospital, Pisa, Italy; 2 Department of Surgical, Medical, Molecular and Critical Area Pathology, University of Pisa, Via Savi, Pisa, 10 - 56126 Italy; 3 Emergency Department, Hospital of Merano, Merano, Italy; 4 Emergency Department, University of Verona, Verona, Italy; 5 Department of Clinical and Experimental Medicine, University of Pisa, Pisa, Italy

**Keywords:** Brain injury, Anticoagulation, Trauma

## Abstract

**Background:**

Nomograms are easy-to-handle clinical tools which can help in estimating the risk of adverse outcome in certain population. This multi-center study aims to create and validate a simple and usable clinical prediction nomogram for individual risk of post-traumatic Intracranial Hemorrhage (ICH) after Mild Traumatic Brain Injury (MTBI) in patients treated with Direct Oral Anticoagulants (DOACs).

**Methods:**

From January 1, 2016 to December 31, 2019, all patients on DOACs evaluated for an MTBI in five Italian Emergency Departments were enrolled. A training set to develop the nomogram and a test set for validation were identified. The predictive ability of the nomogram was assessed using AUROC, calibration plot, and decision curve analysis.

**Results:**

Of the 1425 patients in DOACs in the study cohort, 934 (65.5%) were included in the training set and 491 (34.5%) in the test set. Overall, the rate of post-traumatic ICH was 6.9% (7.0% training and 6.9% test set). In a multivariate analysis, major trauma dynamic (OR: 2.73, p = 0.016), post-traumatic loss of consciousness (OR: 3.78, p = 0.001), post-traumatic amnesia (OR: 4.15, p < 0.001), GCS < 15 (OR: 3.00, p < 0.001), visible trauma above the clavicles (OR: 3. 44, p < 0.001), a post-traumatic headache (OR: 2.71, p = 0.032), a previous history of neurosurgery (OR: 7.40, p < 0.001), and post-traumatic vomiting (OR: 3.94, p = 0.008) were independent risk factors for ICH. The nomogram demonstrated a good ability to predict the risk of ICH (AUROC: 0.803; CI95% 0.721–0.884), and its clinical application showed a net clinical benefit always superior to performing CT on all patients.

**Conclusion:**

The Hemorrhage Estimate Risk in Oral anticoagulation for Mild head trauma (HERO-M) nomogram was able to predict post-traumatic ICH and can be easily applied in the Emergency Department (ED).

## Background

The use of direct oral anticoagulants (DOACs) in the prevention and treatment of thromboembolism and, more recently, in the primary prevention of cardiovascular events, is steadily increasing and gradually replacing older anticoagulant therapies (heparin, Vitamin K Antagonists) or even antiplatelet therapy [1–3]. As a result, an increasing number of patients with a constitutionally higher bleeding risk due to DOACs treatment require a significant diagnostic and therapeutic effort in the ED, even for minor pathological conditions (e.g., minor trauma, non-serious bleeding) [4]. Mild traumatic brain injury (MTBI) represents the most sensitive diagnostic challenge in this category of patients, because of its pathophysiologic features and the frequency of occurrence [5, 6].

Currently, the main existing guidelines for managing head trauma suggested that computerized tomography (CT) should be performed in all MTBI patients with coagulation abnormalities [7–12]. However, several recent studies demonstrated that the rate of post-traumatic intracranial bleeding (ICH) after an MTBI in the patient on DOACs was limited [13–16] and in the absence of risk factors related to the trauma itself, it became close to zero [17–19]. The identification of clinical risk factors aimed at creating a predictive tools was useful in patients treated with DOACs management [14, 20–22]. Even in this population with an apparent increased hemorrhagic risk, standardization of risk factor analysis into precise predictive models could facilitate decision-making for patients with MTBI in DOACs, promoting cost-effective use of available diagnostic resources. However, no specific predictive models are available so far.

Thus, the aim of this study was to use the clinical risk factors for post-traumatic ICH, which were recently confirmed also in patients on DOACs [20, 21], to develop a clinically applicable nomogram for predicting the likelihood of ICH after MTBI in the patient taking DOACs.

## Methods

### Study design and setting

A retrospective observational multi-center study was conducted evaluating all patients with MTBI on DOACs in five Italian EDs between January 1, 2016, and December 31, 2019 (Ospedale Civile Maggiore in Verona, 100,000 visits per year; Policlinico Universitario in Verona, 50,000 visits per year; Policlinico Universitario in Pisa, 90,000 visits per year; Ospedale Generale in Merano, 70,000 visits per year; and Ospedale Generale in San Bonifacio, 60,000 visits per year). The study was conducted with the approval of local ethical committees (Ethics Committee Clinical Experiments of Verona, Italy, approval number 889CESC; Ethics Committee Clinical Experiments of Bolzano, Italy, approval number 75-2019; Ethics Committee Clinical Experiments of Pisa, Italy approval number 11924_CIPRIANO), according to the ethical principles for medical research involving human subjects of the Declaration of Helsinki.

### Patients

The study inclusion criteria were: MTBI defined as any craniofacial district closed trauma with a Glasgow Coma Scale (GCS) of 14–15, independent of the loss of consciousness immediately following the trauma or any neurological deficit related to it [6, 23, 24] and age > 18 years. Exclusion criteria were: access to the ED more than forty-eight hours after the trauma, an ineffective Oral Anticoagulation Therapy (OAT), defined as a last intake of DOACs beyond twenty-four hours before the trauma, and patients transferred from other EDs.

The records of patients on DOACs and MTBI therapy were identified by extraction from the respective computer databases through dedicated management software (FirstSTATA for Verona & Pisa and QlikView for Merano) all patients who performed a head CT in the ED during the study period.

The selection of patients in line with the inclusion and exclusion criteria was performed through manual chart review by a group of Emergency Physicians with more than five years’ experience.

### Variables

We selected the most representative risk factors for ICH, based on the current literature.

The pre-traumatic risk factors are age, concomitant treatment with antiplatelet agents, alcohol or drug intoxication, presence of known disability, history of epilepsy, history of previous neurosurgical intervention, known psychiatric condition, and a major trauma dynamic defined as dangerous mechanism and/or high-energy impact as indicated in ATLS guidelines [8, 10, 11, 25]. The post-traumatic conditions are post-traumatic loss of consciousness, any form of amnesia, post-traumatic vomiting, persistent headache, visible trauma above the clavicle, a GCS of less than 15 at the first evaluation in the ED, other post-traumatic fractures, and post-traumatic seizure [7, 8, 10, 11, 26].

### Outcomes

The finding of post-traumatic ICH in the head CT scan performed on arrival in the ED (immediate) or in the head CT scan performed after 24 h of clinical observation (delayed) was the primary endpoint of the study. CT positivity was considered as the presence of subdural, epidural, or parenchymal hematoma, subarachnoid hemorrhage, or cerebral contusion [6, 23, 24]. Finally, outcomes important to the patient were defined as the need for neurosurgical intervention (craniotomy, craniectomy, placement of a hole or subdural drainage) or death from post-traumatic ICH within 30 days of trauma [6, 23, 24]. Where direct patient reassessment was not possible at 30 days after injury, follow-up was reconstructed by evaluating the medical records available in the computer databases of the EDs in the study, and mortality was confirmed through the registry office.

### Statistical analysis

Continuous variables were described as mean and standard deviation or median and interquartile range depending on the underlying distribution. Categorical variables were described as the percentage and number of events in the total. Differences between categorical variables were tested using Fisher’s Exact tests or with the Chi-square test while t-tests or with the Mann-Whitney test for continuous variables.

To construct and validate the nomogram, the patient cohort was randomly divided into the training (derivation cohort) and test (validation cohort) sets; 2/3 of the cohort was used to develop the prediction model while the remaining 1/3 of the patients were used to validate the model. Any differences in the two cohorts were explored.

The proposed nomogram, HERO-M (Hemorrhage Extimate Risk in Oral Anticoagulation for Mild head trauma) is a tool to estimate the probability of post-traumatic ICH, based on a weighted score of the eight independent prognostic factors for posttraumatic ICH. It was first applied to a single center pilot study in 451 patients and subsequently reworked in this study [19].

To generate the nomogram, the first step was to perform on the training set the univariate analysis of pre- and post-trauma clinical risk factors identified with the risk of post-traumatic ICH. The variables which were found to be significant in the univariate analysis with a significance level of 0.05, were proposed to the multivariate model for the creation of the nomogram. The multivariate model was run with multivariate logistic regression to test the association between the predictor variables and the probability of ICH. The regression coefficients from the multivariable logistic regression model were then used to generate a nomogram predicting the probability of post-traumatic ICH.

The sum of the individual weighted scores for each of the eight independent prognostic factors is used to obtain the “total score,“ which in turn is associated in the probability axis with the individual risk of post-traumatic ICH presented by the patient. A higher calculated total score is associated with a higher probability of post-traumatic ICH. For example, a patient with a post-traumatic TLOC (score 6.5), with a frontal lacerated wound (score 6.5), and with at least one episode of post-traumatic vomiting (score 7) will have a total score of 20, which is associated with a 40% risk of ICH.

Discriminatory ability, the ability of the model to separate patients with different outcomes, and calibration, which is how different the predictions are from the actual outcomes, were analyzed to validate the model on the test cohort. Nomogram discrimination was assessed using the area under the operating characteristic curve (AUROC. Finally, the net clinical benefit of applying the nomogram was also evaluated with decision curve analyses (DCA) where performing the nomogram is compared with the two baseline strategies (perform CT at all, perform CT at none) for different thresholds of the probability of risk of post-traumatic ICH. All tests were two-sided, and P < 0.05 was considered statistically significant. Statistical analyses were performed with Stata® version 16.0 (StataCorp, College Station, Texas, USA).

## Results

### Patients and trauma risk factors

**Fig. 1:**
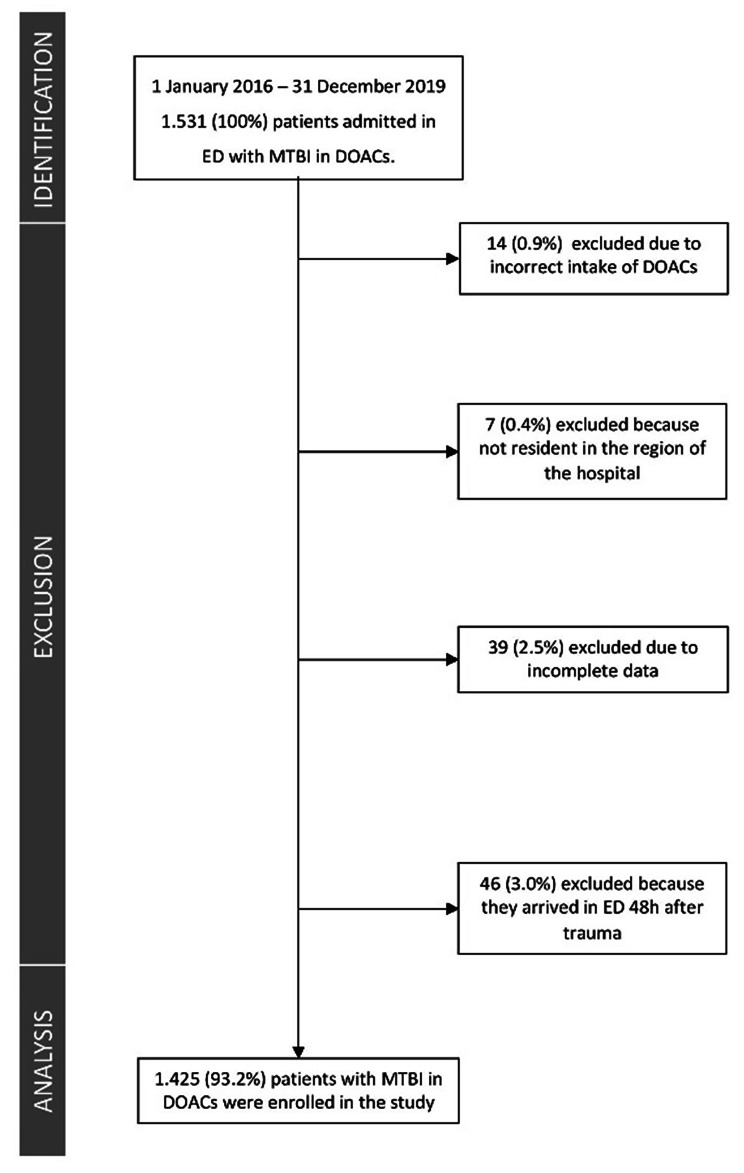
Selection of the study population

Of the 1531 patients on DOACs with MTBI included in the entire study cohort, 1425 were included in the study (Fig. 1). The characteristics of patients included in the training (n = 934) and test (n = 491) sets are reported in Table 1.

**Table 1:** Characteristics of patients with MTBI in DOACs, divided into the derivation and validation cohorts

Variables	Derivation cohort	Validation cohort	p-value
Patients, n (%)	934 (65.5)	491 (34.5)	
**Age, years, median (IQR)**	83 (78–88)	82 (77–87)	0.36
Sex, n (%)			0.50
Male	415 (44.4)	228 (46.4)	
Female	519 (55.6)	263 (53.6)	
**Clinical history, n (%)**			
Hypertension	760 (81.4)	387 (78.9)	0.34
Ischemic heart disease	155 (16.6)	78 (15.9)	0.80
Chronic heart failure	117 (12.5)	77 (15.7)	0.15
Cancer	53 (5.7)	25 (5.1)	0.79
Chronic renal failure	105 (11.2)	43 (8.7)	0.22
Hepatopathy	27 (2.9)	12 (2.5)	1.00
Vasculopathy	106 (11.4)	68 (13.9)	0.28
Diabetes	555 (59.4)	304 (62.0)	0.44
Stroke	157 (16.8)	92 (18.8)	0.41
**Type of DOACs, n (%)**			0.48
Apixiban	333 (35.7)	169 (34.4)	
Dabigatran	200 (21.4)	123 (25.1)	
Edoxaban	92 (9.9)	44 (9.0)	
Rivoroxaban	309 (33.1)	155 (31.6)	
**Indication for DOACs, n (%)**			0.02
Atrial Fibrillation	873 (93.5)	435 (88.7)	
Pulmonary Embolism	42 (4.5)	41 (8.2)	
Others	19 (2.0)	15 (3.1)	
**Trauma dynamics, n (%)**			0.41
Ground falls	664 (71.1)	333 (67.9)	
Other falls	41 (4.4)	28 (5.7)	
Road accident	45 (4.8)	15 (3.1)	
Precipitation	8 (0.9)	4 (0.8)	
Direct trauma	17 (1.8)	13 (2.6)	
Pre-traumatic transitory loss of consciousness	159 (17.0)	98 (20.1)	
**Time elapsed between trauma and CT, n (%)**			0.47
Within 3 hours	479 (51.3)	243 (49.5)	
Between 3 and 8 hours	176 (18.8)	106 (21.6)	
Over 8 hours	279 (29.9)	142 (28.9)	
**Post-traumatic ICH, n (%)**	65 (7.0)	34 (6.9)	1.00

The proportion of patients with a post-traumatic ICH was 7% (65/934) in the training cohort and 6.9% (34/491) in the test cohort, p = 1.000. No differences in patients’ characteristics were recorded between the two patient cohorts. A slight discrepancy was observed in the indication for DOACs.

The univariate analysis of pre- and post-traumatic clinical risk factors with the presence of post-traumatic ICH performed in the derivation cohort is reported in Table 2.

**Table 2:** Pre- and post-traumatic risk factors divided between patients who have and have not reported post-traumatic ICH.

Variables	No ICH	ICH	p-value
**Patients, n (%)**	869 (93.0)	65 (7.0)	
**Age, years, median (IQR)**	83 (78–88)	84 (78–88)	0.318
**Pre-traumatic risk factors, n (%)**			
Major trauma dynamic	29 (3.3)	9 (13.8)	0.001
Acute intoxication	13 (1.5)	0 (0.0)	-
Concomitant antiplatelet therapy	73 (8.4)	5 (7.7)	0.84
Chronic psychiatric disease	60 (6.9)	6 (8.5)	0.56
Chronic cognitive impairment	176 (20.3)	15 (23.4)	0.58
Motor disability	233 (26.8)	24 (36.2)	0.18
Previous neurosurgery	30 (2.3)	12 (18.5)	0.002
History of epilepsy	32 (3.7)	4 (6.4)	0.42
**Post-traumatic risk factor, n (%)**			
Post-traumatic transitory loss of consciousness	30 (3.5)	12 (18.5)	< 0.001
Post-traumatic amnesia	74 (8.5)	21 (32.3)	< 0.001
Evidence of trauma above the clavicles	557 (64.1)	55 (84.6)	0.001
Other post-traumatic fracture	330 (38.0)	17 (26.2)	0.06
GCS < 15	74 (8.5)	14 (21.5)	0.002
Signs of skull base fracture	3 (0.3)	1 (1.5)	0.25
Post-traumatic vomiting	16 (1.8)	7 (10.8)	0.001
Post-traumatic headache	26 (3.0)	7 (10.8)	0.006
Post-traumatic seizure	3 (0.3)	0 (0.0)	-

Pre-traumatic risk factors found to be associated with the presence of post-traumatic ICH were the presence of a major trauma dynamic (13.8% versus 3.3%, p = 0.001) and a previous neurosurgical intervention (12.8% versus 2.5%, p = 0.002). Among the post-traumatic risk factors, those found to be associated with the presence of post-traumatic ICH were a Traumatic Loss Of Consciousness (TLOC; 18.5% versus 3.5%, p < 0.001), post-traumatic amnesia (32.3% versus 8.5%, p < 0.001), evidence of trauma above the clavicles (84. 6% versus 64.1%, p = 0.001), the presence of a GCS less than 15 at the time of the visit evaluation (21.5% versus 8.5%, p = 0.002), at least one episode of post-traumatic vomiting (10.8% versus 1.8%, p = 0.001), and post-traumatic headache (10.8% versus 3%, p = 0.006).

### Model characteristics and predictors of post-traumatic ICH

The pre- and post-traumatic risk factors found to be associated with the presence of post-traumatic ICH based on the previous univariate analysis were candidates for the multivariate predictive model. The multivariate model identified eight risk factors which were found to be associated with the risk of post-traumatic ICH (Table 3): post-traumatic TLOC presented an OR of 3.78 (CI95% 1.78–8.04, p = 0.016), major dynamic presented an OR of 2.73 (CI95% 1.21–6.18, p = 0. 001), presence of post-traumatic amnesia reported an OR of 4.15 (CI95% 2.37–7.27, p < 0.001), GCS less than 15 with an OR of 3.00 (CI95% 1.65–5.47, p < 0.001), visible trauma above the clavicles with an OR of 3. 44 (CI95% 1.87–6.33, p < 0.001), a post-traumatic headache with an OR found to be equal to 2.71 (CI95% 1.09–6.73, p = 0.032) also a previous history of neurosurgery reported an OR equal to 7.40 (CI95% 3.29–16.65, p < 0. 001), finally post-traumatic vomiting reported an OR of 3.94 (CI95% 1.44–10.82, p = 0.008).

**Table 3:** Multivariate analysis of all risk factors found significant for post-traumatic ICH at the previous univariate analysis

	Odds Ratio	95% Confidence Interval	p-value
**Major trauma dynamic**	2.73	1.21–6.18	0.02
**Post-traumatic loss of consciousness**	3.78	1.78–8.04	0.001
**Post-traumatic amnesia**	4.15	2.37–7.27	< 0.001
**GCS < 15**	3.00	1.65–5.47	< 0.001
**Evidence of trauma above the clavicles**	3.44	1.87–6.33	< 0.001
**Post-traumatic headache**	2.71	1.09–6.73	0.03
**Previous neurosurgery**	7.40	3.29–16.65	< 0.001
**Post-traumatic vomiting**	3.94	1.44–10.82	0.01

### Nomogram and validation estimates

**Fig. 2:**
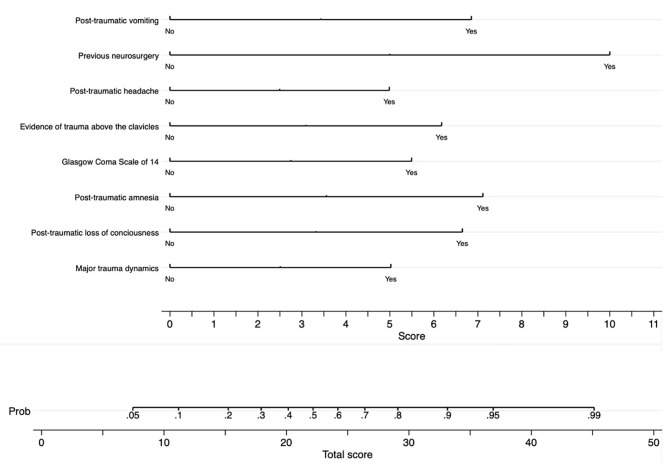
HERO-M nomogram to predict the risk of ICH after mild traumatic brain injury in patients in DOACs.

HERO-M (Hemorrhage Extimate Risk in Oral anticoagulation for Mild head trauma) is a nomogram based on multivariate coefficients to estimate the individual probability of post-traumatic ICH (Fig. 2).

The nomogram is automatically created by assigning a weighted score according to the regression coefficients of the eight independent prognostic factors for post-traumatic ICH (Fig. 2).

**Fig. 3:**
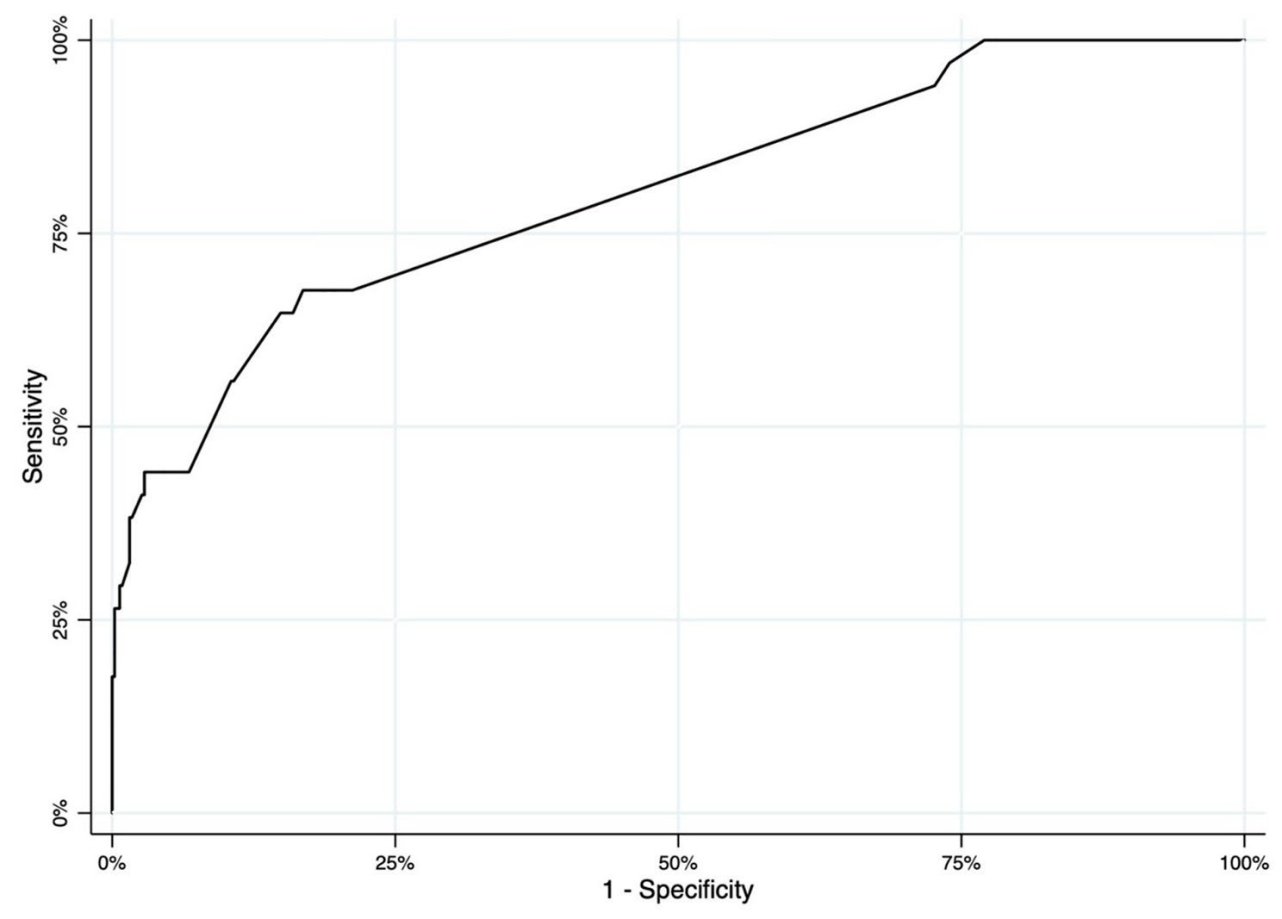
ROC curve of the predictive ability of the HERO-M nomogram towards the risk of ICH.

**Fig. 4:**
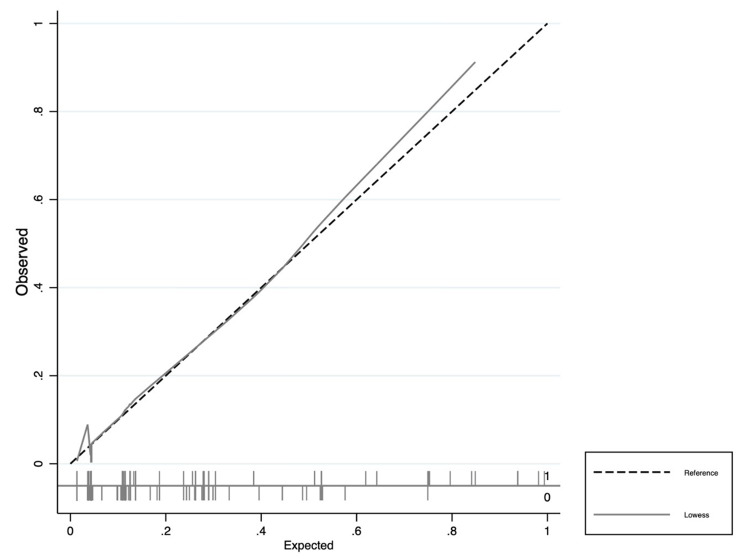
Calibration plot of the HERO-M nomogram in the ICH prediction

**Fig. 5:**
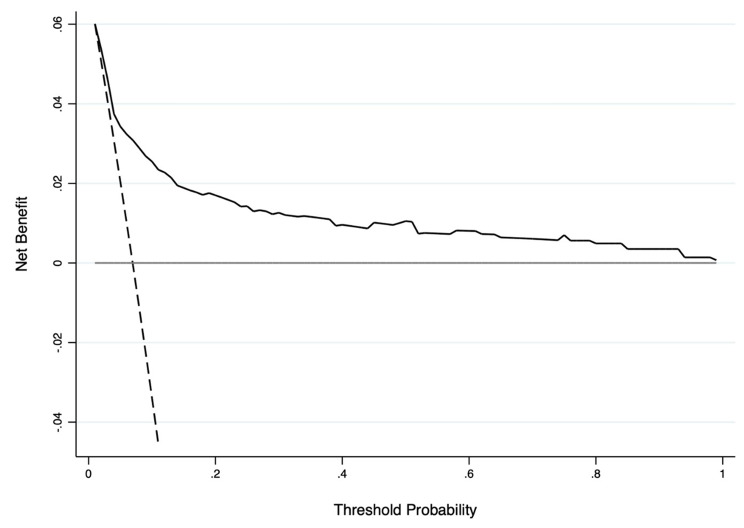
Decision curve analysis of the HERO-M nomogram. The grey line represents the strategy of not performing head CT on any patient, the dashed black line represents the strategy of performing head CT on all while the solid black line represents the execution of the nomogram. The image demonstrates a better discriminatory ability of the nomogram compared to the other two strategies at any threshold probability level

**Fig. 6:**
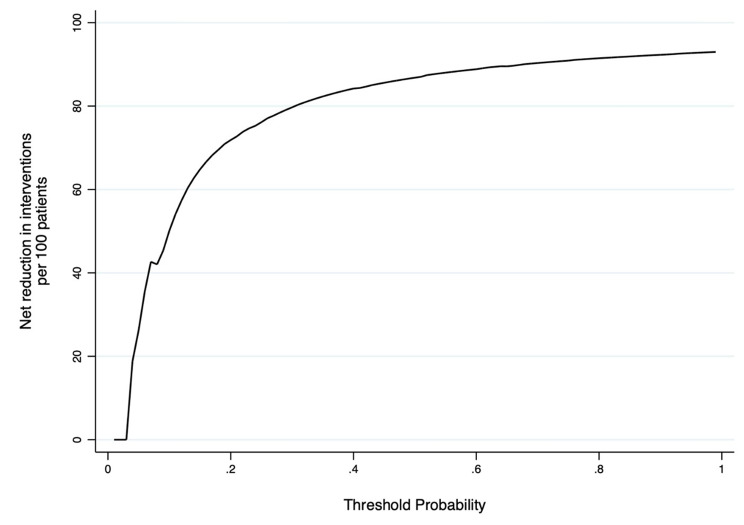
Decision curve analysis plotting the decrease in head CTs due to the application of the HERO-M nomogram based on the prevalence of ICH in the population

The discrimination, calibration, and any net clinical benefit of HERO-M were tested. The discriminatory ability of the nomogram was found to be high, presenting an AUC of 0.803 (CI95% 0.721–0.884) (Fig. 3). The calibration plot revealed a fair model fit predicting the risk of post-traumatic ICH (Fig. 4). Finally, the DCAs demonstrate that the application of the nomogram has a net clinical benefit that is always greater than the strategies of performing CT on all the patients and performing CT on none of the patients (Fig. 5). Despite the small benefit, still superior to performing CT at all, the application of HERO-M would allow a reduction of a good number of CTs within a hypothetical risk of ICH of less than 20% (Fig. 6).

## Discussion

OAT is considered a risk factor for bleeding complications by several international guidelines [7–11]. For this reason, a well-established clinical practice is to perform a head CT scan in all anticoagulated patients with MTBI. NICE guidelines [7] and many of the clinical decision rules (e.g., The Canadian CT Head Rule) confirm the indication to necessarily submit the anticoagulated patient with MTBI to at least one head CT investigation to rule out the risk of ICH even if neurologically intact [6]. Despite prior studies and meta-analyses demonstrating an association between post-traumatic ICH and anticoagulation or treatment with antiplatelet agents in patients with head trauma [22, 27–29], some recent studies did not show such an association [13, 14, 16, 30]. These new observations could be partly due to the different cohorts of patients. In fact, prior studies about TBI in OAT comprehended a more heterogeneous casuistry concerning age and dynamics of trauma, whereas recent literature is more often focused on minor events (i.e. low energy falls) in older adults. Confirming this, as part of the MTBI, Alrajhi et al. found that the risk of ICH dropped to one-fifth in the case of minimal trauma [31]. Kuczawski and colleagues [15] observed in a multi-center observational study how a CT scan for all anticoagulated patients with a head injury is not cost-effective, since only 7 of 1420 (0.5%) who did not receive a CT scan experienced a potentially preventable adverse outcome related to head injury (death from ICH, n = 4; reattendance for ICH, n = 3) .

Despite a reduced rate of post-traumatic ICH, previous indications for the management of patients treated with Vitamin K Antagonists (VKAs) were translated to patients on DOACs. In addition, recent indications seem to suggest that the absence of all clinical risk factors associated with the presence of post-traumatic ICH, significantly reduces the risk of actual ICH and even minimizes the need for neurosurgical intervention also in patients in DOACs, as also previously reported for VKA [6, 20, 21]. However, no predictive or risk modeling system has been proposed to improve patient management in DOACs despite widely emphasized in the literature the need to fill this gap [6, 20].

The present study, for the first time, attempted to model the individual risk of ICH in MTBI patients in DOACs from the risk factors studied for years at the first evaluation in the ED. HERO-M nomogram, in addition to providing in a simple, rapid, and repeatable of identification of ICH risk based on the patient’s clinical phenotype, could also allow a management modulation of the patient in DOACs according to the characteristics presented at access in ED.

Our study considered the creation of the nomogram. Most of the clinical variables that have been studied and included in the different clinical decision rules over the decades to assess the risk of ICH in the patient with MTBI [11, 26, 32]. These risk factors have recently been explored and confirmed as individual risk predictors also in patients with OAT, supporting the hypothesis to include them in a broader predictive model of post-traumatic hemorrhage. Evidence of trauma above the clavicles or the presence of major trauma dynamics seems to be warning signs that could reveal to the clinician the severity of the impact [33]. Despite different and not unanimous definitions of major trauma dynamics, the indication of high-energy trauma is universally recognized as having a poor prognosis even in the small series of the case published so far on DOACs [14, 19]. Certainly, improving the characterization of impact and trauma dynamics could also benefit the calculation of the risk of adverse outcomes. Among the post-traumatic factors found to be significantly associated with the presence of post-traumatic ICH, post-traumatic amnesia resulted the most significant one in in our previous observation [21]. Recent evidence through decision tree analysis observed that post-traumatic amnesia was the most impacting factor representing the root node of the decision tree of patients in DOACs [20]. The absence of acute post-traumatic neurologic symptoms, as the absence of concussion (post-traumatic amnesia, post-traumatic TLOC, GCS within limits) and potentially related symptoms (headache, vomiting), greatly reduces the risk of post-traumatic bleeding even during oral anticoagulation [6, 20, 21]. In agreement with this, Fuller and colleagues raised the doubt that in the absence of objectifiable neurological changes, DOACs therapy alone may not be a sufficient predictor of ICH, so performing head CT in all anticoagulated patients who are victims of MTBI appears to be inappropriate and not cost-effective [17].

The availability of a prediction tool such as the HERO-M nomogram, which unifies the risk provided by each clinical factor into a total individualized estimation, could give to the emergency physician the possibility of modulating patient management on the actual risk presented. Although there is not yet sufficient evidence of safety to change current strategies focused on extensive CT use, this predictive model could at least suggest different ways of subsequent management (observation or discharge of the patient from the ED) in case of negative CT imaging. A future validation of the nomogram could allow to define and create different management pathways based on the various score ad cut-offs.

The application of this tool could allow for a reduction in costs given a minor number of CTs performed, as well as a greater availability of devices for other types of emergencies at the ED. The advantage for the patient would be a lower exposure to X-rays and the reduction of waiting times in the ED, correlated above all for the elderly with disorientation and delirium, symptoms that could irreparably confuse the clinical picture [34]. Nevertheless, a longer period of observation is not required, since the percentage of delayed complications are quite rare and usually accompanied by symptoms or signs [21].

We are aware, however, that only precise, replicable, and safe risk modeling can move away from such an unsuccessful strategy as extensive CT use and that prospective studies in this direction are urgently needed in the near future.

### Limitations

Firstly, the study presents the typical biases of the observational retrospective nature, including a selection defect, due to errors or omissions in documentation. However, the common clinical practice of the identification of widely known risk factors made it possible to limit some of the biases. Second, an accurate sample calculation was not conducted due to the absence of a large number of patients. However, the course of the present study remains very large compared with those published so far, and the method of dividing the cohort into two training and validation data sets also allowed a correct methodology to be performed in evaluating the effectiveness of the nomogram. Third, not all patients repeated a follow-up CT scan at the end of the follow-up period. However, all enrolled patients were re-evaluated 30 days after trauma, which made it possible to exclude the occurrence or evolution of clinically significant hemorrhagic complications. Fourth, a priori choice of risk factors was performed (e.g., major trauma dynamics). Fifth, the validation technique performed is a virtual validation that does not make possible to really evaluate patients who have led to a not perfect AUROC, therefore further validation studies with external cohorts will be necessary. However, the performance of AUROC may be linked to increased CT performance in patients with premonitory signs but who later report a negative CT, compared to patients who do not perform CT and who subsequently have an ICH. Finally, the dosages of DOACs were not available, and the analyses were not mediated for the dosage of DOACs.

## Conclusions

In summary, the risk nomogram seems to be a tool that easily and quickly can provide an estimate of the risk of post-traumatic ICH based on the patient’s clinical condition. As the validation analyses demonstrate, it seems to possess good discriminatory abilities and provide a net clinical benefit, especially for low-risk patients where it could allow, if further validation is done, to avoid the extensive use of CT.

## Data Availability

The datasets used and analyzed during the current study are available from the corresponding author on reasonable request.

## List of Abbreviations

CT: Computed tomography
DCA: Decision curve analysis
DOACs: Direct oral anticoagulants
ED: Emergency department
GCS: Glasgow coma scale
ICH: Intracranial hemorrhage
MTBI: Mild traumatic brain injury
OAT: Oral anticoagulation therapy
TBI: Traumatic brain injury
TLOC: Traumatic loss of consciousness
VKAs: Vitamin K antagonists

## References

[1] Khan SU, Khan MZ, Asad ZUA, Valavoor S, Khan MU, Khan MS (2020). Efficacy and safety of low dose rivaroxaban in patients with coronary heart disease: a systematic review and meta-analysis. J Thromb Thrombolysis.

[2] Kearon C, Akl EA, Ornelas J, Blaivas A, Jimenez D, Bounameaux H (2016). Antithrombotic therapy for VTE Disease: CHEST Guideline and Expert Panel Report. Chest.

[3] Tang T, Zhang M, Li W, Hu N, Du X, Ran F (2021). Oral anticoagulant and antiplatelet therapy for peripheral arterial disease: a Meta-analysis of Randomized controlled trials. Clin Appl thrombosis/hemostasis: Official J Int Acad Clin Appl Thrombosis/Hemostasis.

[4] Maegele M, Grottke O, Schochl H, Sakowitz OA, Spannagl M, Koscielny J (2016). Direct oral anticoagulants in emergency trauma admissions. Deutsches Arzteblatt International.

[5] Garra G, Nashed AH, Capobianco L (1999). Minor head trauma in anticoagulated patients. Acad Emerg Medicine: Official J Soc Acad Emerg Med.

[6] Fuller G, Sabir L, Evans R, Bradbury D, Kuczawski M, Mason SM (2020). Risk of significant traumatic brain injury in adults with minor head injury taking direct oral anticoagulants: a cohort study and updated meta-analysis. Emerg Med J.

[7] Davis T, Ings A, Care E, National Institute of H (2015). Head injury: triage, assessment, investigation and early management of head injury in children, young people and adults (NICE guideline CG 176). Archives of Disease in Childhood Education and Practice Edition.

[8] Vos PE, Battistin L, Birbamer G, Gerstenbrand F, Potapov A, Prevec T (2002). EFNS guideline on mild traumatic brain injury: report of an EFNS task force. Eur J Neurol.

[9] Firsching R, Rickels E, Mauer UM, Sakowitz OW, Messing-Junger M, Engelhard K (2017). Guidelines for the treatment of Head Injury in adults. Journal of neurological surgery part A. Cent Eur Neurosurg.

[10] Mower WR, Hoffman JR, Herbert M, Wolfson AB, Pollack CV, Zucker MI (2002). Developing a clinical decision instrument to rule out intracranial injuries in patients with minor head trauma: methodology of the NEXUS II investigation. Ann Emerg Med.

[11] Smits M, Dippel DW, Steyerberg EW, de Haan GG, Dekker HM, Vos PE (2007). Predicting intracranial traumatic findings on computed tomography in patients with minor head injury: the CHIP prediction rule. Ann Intern Med.

[12] Haydel MJ, Preston CA, Mills TJ, Luber S, Blaudeau E, DeBlieux PM (2000). Indications for computed tomography in patients with minor head injury. N Engl J Med.

[13] Galliazzo S, Bianchi MD, Virano A, Trucchi A, Donadini MP, Dentali F (2019). Intracranial bleeding risk after minor traumatic brain injury in patients on antithrombotic drugs. Thromb Res.

[14] Nishijima DK, Gaona SD, Waechter T, Maloney R, Blitz A, Elms AR (2018). The incidence of traumatic intracranial hemorrhage in Head-Injured older adults transported by EMS with and without anticoagulant or antiplatelet use. J Neurotrauma.

[15] Kuczawski M, Stevenson M, Goodacre S, Teare MD, Ramlakhan S, Morris F (2016). Should all anticoagulated patients with head injury receive a CT scan? Decision-analysis modelling of an observational cohort. BMJ open.

[16] Uccella L, Zoia C, Bongetta D, Gaetani P, Martig F, Candrian C (2018). Are Antiplatelet and Anticoagulants Drugs a risk factor for bleeding in mild traumatic brain Injury?. World Neurosurg.

[17] Fuller GW, Evans R, Preston L, Woods HB, Mason S (2019). Should adults with mild Head Injury who are receiving direct oral anticoagulants undergo computed tomography scanning? A systematic review. Ann Emerg Med.

[18] Nederpelt CJ, van der Aalst SJM, Rosenthal MG, Krijnen P, Huisman MV, Peul WC (2020). Consequences of pre-injury utilization of direct oral anticoagulants in patients with traumatic brain injury: a systematic review and meta-analysis. J Trauma Acute care Surg.

[19] Turcato G, Zannoni M, Zaboli A, Zorzi E, Ricci G, Pfeifer N et al. Direct oral anticoagulant treatment and mild traumatic brain Injury: risk of early and delayed bleeding and the severity of injuries compared with vitamin K antagonists. The Journal of emergency medicine. 2019. 10.1016/j.jemermed.2019.09.007 31648805

[20] Turcato G, Zaboli A, Pfeifer N, Maccagnani A, Tenci A, Giudiceandrea A (2021). Decision tree analysis to predict the risk of intracranial haemorrhage after mild traumatic brain injury in patients taking DOACs. Am J Emerg Med.

[21] Cipriano A, Park N, Pecori A, Bionda A, Bardini M, Frassi F (2021). Predictors of post-traumatic complication of mild brain injury in anticoagulated patients: DOACs are safer than VKAs. Intern Emerg Med.

[22] Riccardi AGG, Chiarbonello B, Frumento F, Polletti P, Castelli M, Minuto P, Lerza R (2014). Minor head injury in anticoagulated patients: a 6-year retrospective analysis in an emergency department. Emerg Care J.

[23] Servadei F, Teasdale G, Merry G, Neurotraumatology Committee of the World Federation of Neurosurgical S (2001). Defining acute mild head injury in adults: a proposal based on prognostic factors, diagnosis, and management. J Neurotrauma.

[24] Easter JS, Haukoos JS, Meehan WP, Novack V, Edlow JA (2015). Will Neuroimaging reveal a severe Intracranial Injury in this adult with minor Head Trauma?: the rational clinical examination systematic review. JAMA.

[25] American College of Surgeons’ Committee on T. Advanced Trauma Life Support® Student Course Manual Library of Congress Control Number: 20179079972018.

[26] Alzuhairy AKA (2020). Accuracy of canadian CT Head Rule and New Orleans Criteria for Minor Head Trauma; a systematic review and Meta-analysis. Archives of Academic Emergency Medicine.

[27] Minhas H, Welsher A, Turcotte M, Eventov M, Mason S, Nishijima DK (2018). Incidence of intracranial bleeding in anticoagulated patients with minor head injury: a systematic review and meta-analysis of prospective studies. Br J Haematol.

[28] Grandhi R, Harrison G, Voronovich Z, Bauer J, Chen SH, Nicholas D (2015). Preinjury warfarin, but not antiplatelet medications, increases mortality in elderly traumatic brain injury patients. J Trauma Acute care Surg.

[29] Jeffree RL, Gordon DH, Sivasubramaniam R, Chapman A (2009). Warfarin related intracranial haemorrhage: a case-controlled study of anticoagulation monitoring prior to spontaneous subdural or intracerebral haemorrhage. J Clin Neuroscience: Official J Neurosurgical Soc Australasia.

[30] Lampart A, Kuster T, Nickel CH, Bingisser R, Pedersen V (2020). Prevalence and severity of traumatic intracranial hemorrhage in older adults with Low-Energy Falls. J Am Geriatr Soc.

[31] Alrajhi KN, Perry JJ, Forster AJ (2015). Intracranial bleeds after minor and minimal head injury in patients on warfarin. J Emerg Med.

[32] Stiell IG, Lesiuk H, Wells GA, McKnight RD, Brison R, Clement C (2001). The canadian CT Head Rule Study for patients with minor head injury: rationale, objectives, and methodology for phase I (derivation). Ann Emerg Med.

[33] Molaei-Langroudi R, Alizadeh A, Kazemnejad-Leili E, Monsef-Kasmaie V, Moshirian SY (2019). Evaluation of clinical criteria for performing brain CT-Scan in patients with mild traumatic Brain Injury; a New Diagnostic Probe. Bull Emerg Trauma.

[34] Chen F, Liu L, Wang Y, Liu Y, Fan L, Chi J (2022). Delirium prevalence in geriatric emergency department patients: a systematic review and meta-analysis. Am J Emerg Med.

